# The Genome in a Three-Dimensional Context: Deciphering the Contribution of Noncoding Mutations at Enhancers to Blood Cancer

**DOI:** 10.3389/fimmu.2020.592087

**Published:** 2020-10-07

**Authors:** Llorenç Rovirosa, Alberto Ramos-Morales, Biola M. Javierre

**Affiliations:** ^1^ 3D Chromatin Organization Group, Josep Carreras Leukaemia Research Institute (IJC), Germans Trias i Pujol, Badalona, Spain; ^2^ Institute for Health Science Research Germans Trias i Pujol (IGTP), Badalona, Spain

**Keywords:** spatial genome architecture, 3D chromatin organization, DNA loops, noncoding mutations and epimutations, enhancers, blood cancer, hematopoietic malignancies

## Abstract

Associations between blood cancer and genetic predisposition, including both inherited variants and acquired mutations and epimutations, have been well characterized. However, the majority of these variants affect noncoding regions, making their mechanisms difficult to hypothesize and hindering the translation of these insights into patient benefits. Fueled by unprecedented progress in next-generation sequencing and computational integrative analysis, studies have started applying combinations of epigenetic, genome architecture, and functional assays to bridge the gap between noncoding variants and blood cancer. These complementary tools have not only allowed us to understand the potential malignant role of these variants but also to differentiate key variants, cell-types, and conditions from misleading ones. Here, we briefly review recent studies that have provided fundamental insights into our understanding of how noncoding mutations at enhancers predispose and promote blood malignancies in the context of spatial genome architecture.

## Introduction: Why We Need to Study Enhancers and Spatial Genome Organization to Understand Blood Cancer

Noncoding regions of the genome, which comprise more than 98% of the genome, have historically been overlooked. However, more than 95% of the risk variants associated with genetically complex diseases, such as blood cancer, map at noncoding regions and remain unexplored due to the lack of an obvious disease mechanism (1). In addition, the oncogenic potential of somatically acquired noncoding mutations has become increasingly evident (2). The mutational rate of the noncoding genome is significantly higher than its coding counterpart, although the selective pressure is weaker unless the mutations have a functional effect that confers an advantage to cell survival (3). For this reason, in the search for noncoding mutations it is important to find and focus on functional regions that impact gene regulation such as enhancers.

Enhancers are frequent targets of genetic and epigenetic alterations in many diseases including blood cancer (1, 4–6). The human genome contains around one million enhancers, many of which are cell-type or stimulus-specific (7). They modulate the activation of promoters and fine-tune transcription over large genomic distances, independent of sequence orientation and position (8). It is now generally accepted that long-range enhancers are brought into close spatial proximity with the promoters they regulate. This proximity is determined by the DNA folding into loops, which are cell-type and stimulus-specific (6, 9–11).

**Figure 1:**
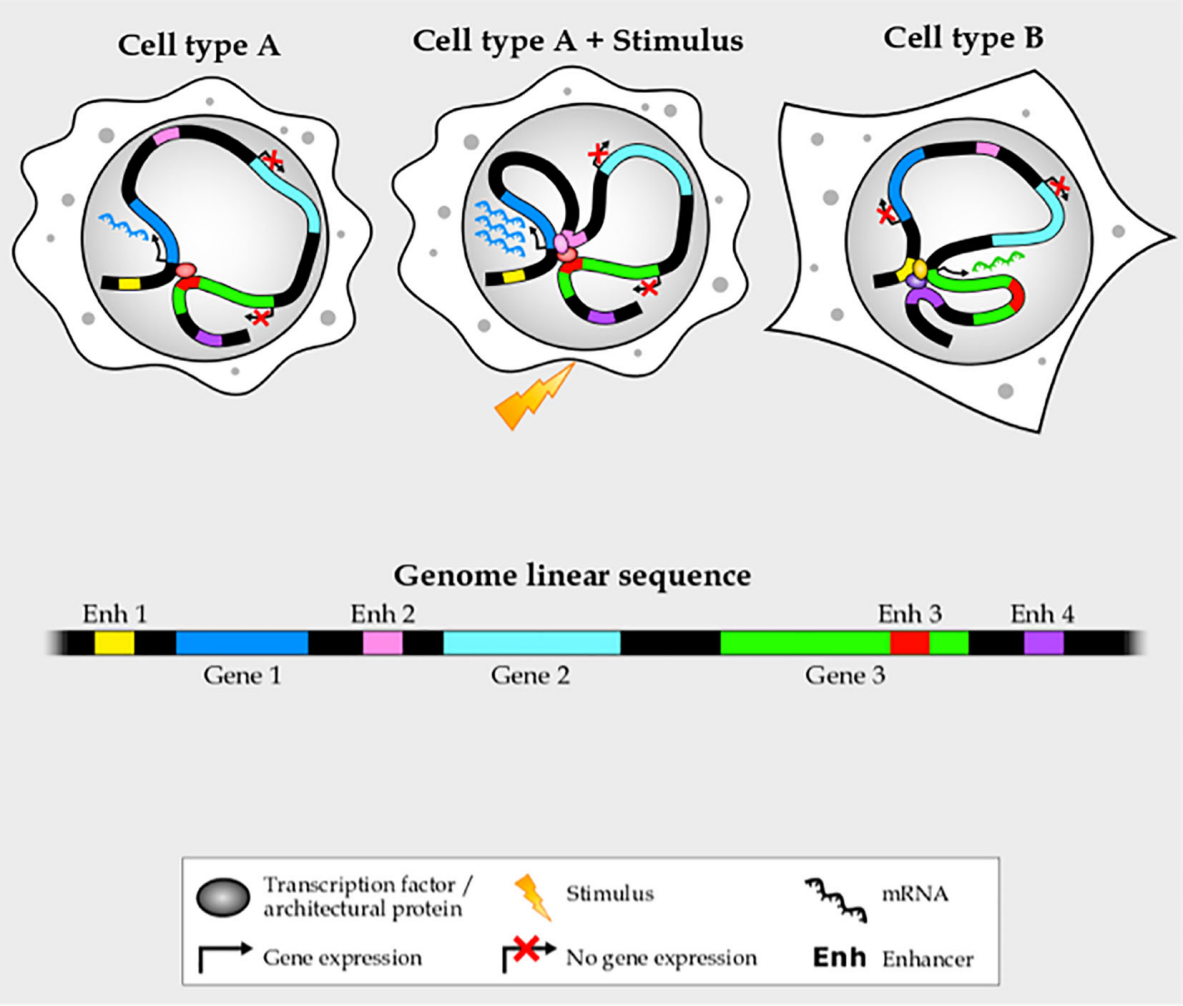
Enhancer–promoter interactions are complex and cell- and stimulus-specific.

Though enhancers have been relatively easy to identify—since they harbor a high density of DNA motifs recognized by transcription factors (12–14), have specific histone modification and co-factor binding profiles (13, 15–17), and produce enhancer RNAs (18, 19) —their target genes are harder to pinpoint (7, 20). Enhancers can be up to a few megabases away from their targets, often jumping over several intervening genes or even being located in the intron of a non-target gene (21). In addition, a given gene is frequently controlled by more than one enhancer, and an enhancer can control more than one gene (22, 23) (**Figure 1**). Enhancers cannot simply be assigned to the nearest gene, and most of their target genes in each cell-type and stimulus condition remain unknown. For this reason, during the last years chromatin conformation capture (3C) techniques, such as Hi-C (24), a technique designed to identify the entire ensemble of chromosomal interactions within a cell population, have emerged to support that spatio-temporal chromatin organization not only plays a key role in the function of enhancers, but will also help scientists identify target genes, thus helping us determine the disease mechanism of enhancer mutations.

In the following sections, we will review the current knowledge on the effect of noncoding mutations on enhancers and genomic 3D structure in blood malignancies. We will highlight the cognate clinical implications, not only in better understanding oncogenesis, but also in the identification of potential new biomarkers and therapeutic strategies to improve disease diagnosis, monitoring and treatment.

## The Three-Dimensional Context of the Genome Plays a Major Role in the Development and Progression of Blood Cancer

Chromatin interactions are crucial for cellular health, and errors in these interactions give rise to a broad range of diseases, including blood cancers (25–27). Cancer-associated alterations in chromatin architecture increase proliferation and decrease differentiation capacity by blocking cell differentiation, altering the expression of oncogenic or tumor suppressor genes, and/or creating unnatural cell states with deregulated expression of key developmental genes. Genomic regions brought into close proximity by the chromatin architecture have a higher frequency of producing translocations, thus explaining the frequency of recurrent pathological translocations such as BCR-ABL and MYC-IGH (26, 27). In addition to chromosome-scale alterations, three-dimensional chromatin organization works on a smaller scale to impact gene regulation by rewiring physical interactions between gene promoters and regulatory elements. This important spatial organization is often altered in blood cancer through the genetic alteration or altered binding of the proteins involved in establishing and maintaining chromatin loops.

Architectural proteins, including CTCF and cohesins, play an important role in establishing and maintaining chromatin loops (28, 29), facilitating communication of gene promoters with some regulatory elements while reducing contacts with others (30–32). Cohesin complex mutations, which impair hematopoietic progenitor differentiation (33–35), occur in ~13% of acute myeloid leukemia (AML) patients (36–38). CTCF generally only binds non-methylated DNA sequences (39–42), so cancer-related methylation changes can impact genomic architecture broadly or at specific loci, although in some sites CTCF binding remains independent of DNA methylation suggesting that methylation may be one of many synergistic factors impacting transcription factor binding (43). A recent study by Kloetgen and colleagues characterized global alterations in genome architecture in T-cell precursor acute lymphoblastic leukemia (T-ALL) (44). The authors found that the lack of CTCF-mediated insulation at a specific locus allowed the cancer-driver gene *MYC* to come into direct contact with a distal super-enhancer, thus increasing this transcription factor’s expression, allowing MYC to turn on signal transduction pathways leading to cell growth and proliferation.

Interestingly, altered DNA methylation at enhancers in cancer is more closely related to changes in gene expression than at promoters (45). Hypomethylated enhancers bind transcription factors better than methylated ones (46–49), thus promoting gene expression and potentially affecting genome architecture. Transcription factors can also influence spatial chromatin organization, from ubiquitously expressed factors such as YY1 (50) to cell-type specific factors including GATA1, LBD1, HSPs, KLF4, LacI, MYOD, and OCT4 (51–56). In addition to mutating these proteins, genetic or epigenetic alterations can have profound effects on chromatin architecture by affecting the binding of these structural proteins (48, 57, 58).

Acquired mutations, regardless of whether they directly affect transcription, can alter spatial chromatin organization and chromatin states to ultimately promote cancer-specific transcriptional programs. This complex interplay is nicely deciphered by Yun and colleagues who used an allelic series of mutant mice to model normal, pre-malignant and AML states using the commonly co-occurring *FLT3* and *NPM1* mutations. Each mutation in isolation altered chromatin state, but together, they synergized to cause global alterations in spatial chromatin organization that produced a leukemia-associated transcriptional program (59), despite these mutations lacking direct influence on transcription or epigenetics. For this reason, the development of therapeutic approaches against mutations also needs to take into account cancer-associated genome-wide epigenetic and conformational landscapes. Interestingly, some anti-leukemic drugs partially reverse the altered topology of some genomic regions. For example THZ1, a covalent CDK7 inhibitor, has been shown to abrogate *MYC*-enhancer aberrant contacts (44) and deter growth in T-ALL cell lines (60), potentially accounting for the anti-tumoral effect of these small-molecule inhibitors.

Although we have just started understanding the role of spatial-temporal genome architecture in blood cancer, there is no doubt about the potential therapeutic opportunities it encompasses. In order to understand changes in genomic spatial architecture, we must first understand the causal changes in the underlying DNA, resulting from both inherited variants and somatic mutations.

## Inherited Genetic Susceptibility to Blood Malignancy From the Three-Dimensional Perspective

Blood cancer is not entirely explained by acquired genomic rearrangements, amplifications, deletions or mutations. In fact, inherited genetic predisposition plays a key role (61–63). In fact, survival rates and phenotype differ between racial and ethnic groups (64–66). The effect of genetic background in blood malignancies has been elegantly demonstrated by a recent work from Young and colleagues in which they cross the *Mll-AF9* knockin mouse with differing inbred strains (67). The authors show that genetic background not only impacts blood composition and survival rates, but also the type of malignancy, from ALL to AML or mixed phenotype acute leukemia.

**Figure 2:**
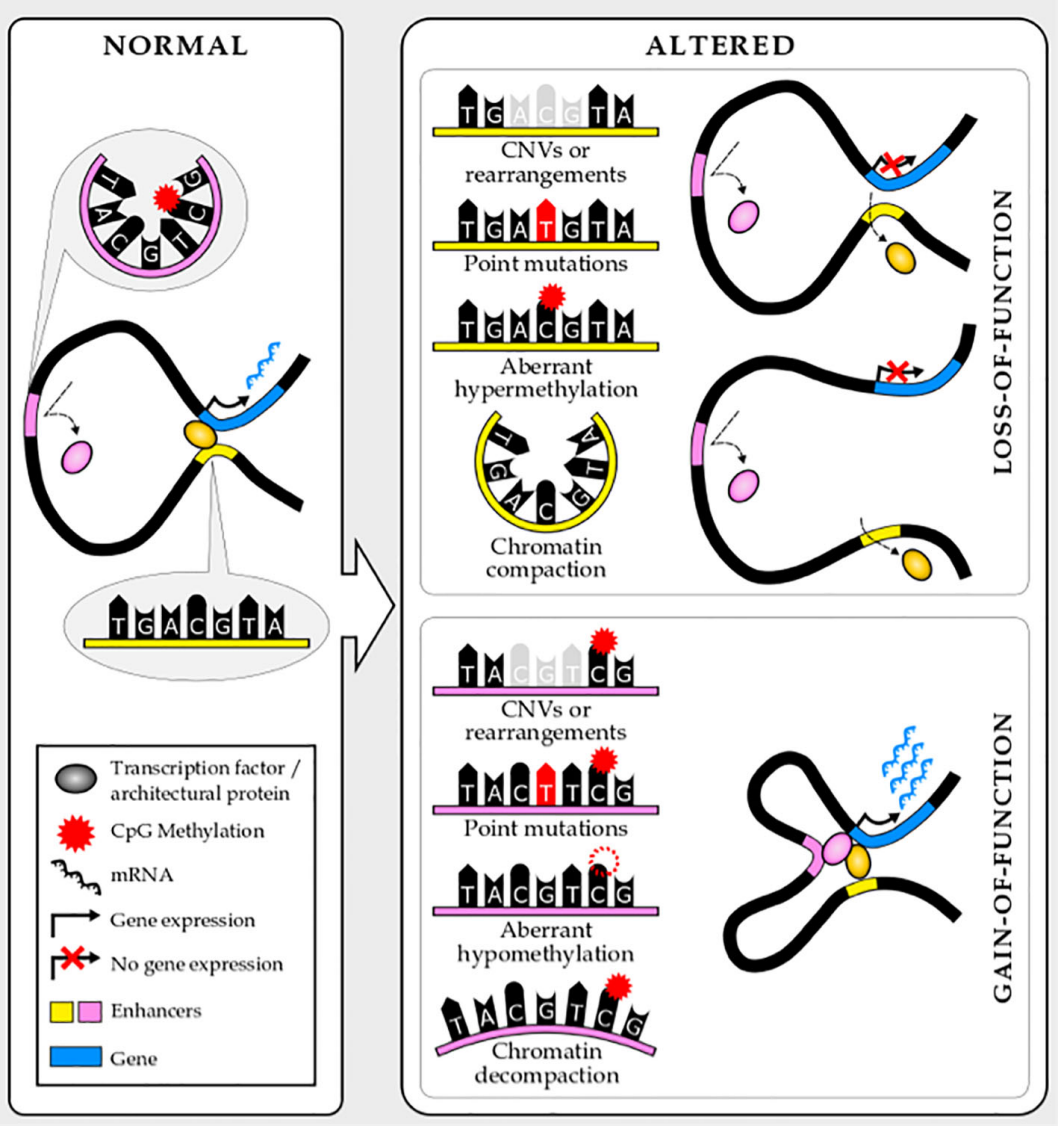
Noncoding mutations and epimutations lead to enhancer loss- or gain-of-function.

Risk loci frequently span noncoding regions (6, 68) and, in fact, cluster at enhancers (1). The single-nucleotide polymorphisms (SNPs) or structural variants (SVs) can alter the binding of transcription factors or structural proteins to regulatory elements by modifying recognition motifs or modulating accessibility, thus altering the expression of their target genes (**Figure 2**). These inherited variants may either disrupt enhancer function, leading to reduced or lost target gene expression, or increase enhancer function, leading to increased target gene expression or even ectopic expression (5). In both cases, if the binding of structural proteins is affected, the enhancer–promoter interaction frequency shifts. However, if transcription factor binding is altered, chromatin looping can either be compromised or not.

Demonstrating this, Speedy and colleagues found that noncoding genetic variants associated with chronic lymphocytic leukemia (CLL) predisposition map to CLL active chromatin, with evidence of that chromatin state being CLL-specific or differentially regulated in normal B-cell differentiation (69). The risk alleles disrupt SPI1, NFKB, and PAX5 binding motifs, or affect accessibility, suggesting a potential impact on enhancer activity. Using chromatin conformation data in parallel with expression data, they connected variants at enhancers with target gene promoters and observed that candidate genes are involved in B-cell biology including immune response, Wnt signaling and apoptosis. Although experimental validation is needed to completely understand the molecular mechanism by which CLL germline variants contribute to malignant transformation, this study underlines the value of epigenetics and genome architecture in interpreting genetic susceptibility.

The mechanistic insights acquired from inherited variants can be extrapolated to acquired mutations and epimutations. Indeed, germline risk variants co-segregate with acquired genomic abnormalities, although the interplay between these remains poorly understood (70, 71). A possible mechanism is that inherited variants alter the expression of pioneer transcription factors, which could synergize with the action of driver mutations to promote genome-wide gene deregulation and oncogenesis. Supporting this hypothesis, Yang and colleagues identified a germline variant at the *GATA3* intron that is strongly associated with Philadelphia-like ALL (72). This enhancer gain-of-function mutation upregulates GATA3 expression, which in turn, reprograms spatial genome architecture and chromatin accessibility genome-wide. This reprogramming puts the oncogene *CRLF2* in proximity to a distal enhancer, promoting CRLF2-mediated constitutive activation of the JAK-STAT pathway, which has been implicated in leukemogenesis (73). This variant is not enough to cause cancer on its own, but it sets the stage for a driver mutation to tip the scales. Not only does GATA3 overexpression facilitate enhancer hijacking by oncogenes, but the authors propose that it also causes chromosomal instability and translocations, due to GATA3 binding sites clustering near Philadelphia-like ALL translocation breakpoints.

## Acquired Mutations During Blood Malignancy Transformation Frequently Target Noncoding Regulatory Elements

Similar to inherited genetic variants, acquired mutations commonly target enhancers, altering gene expression, in blood cancer (74). Mutations preferentially cluster at regulatory elements characteristic to the cell-type from which the tumor originates (75). These mutations may be enhancer gain-of-function, enhancer loss-of-function or enhancer hijacking. Of note, the last category can also be considered a gain-of-function and is frequently linked with major chromosomal rearrangements or insulator alteration (76, 77). Similar to mutations, epimutations such as DNA methylation alteration, also target enhancers (45), and in principle, can affect enhancers in similar ways (**Figure 2**). Silencers and other distal cis-regulatory elements can also be targeted by mutations. However, the narrow characterization of these elements and the incomplete knowledge of their mechanism of action limit their study. Nevertheless, breakthroughs in genomics and molecular biology will put into play these elements in cancer genomics very soon.

Many noncoding gain-of-function mutations, including copy number variation (duplications and deletions) and point mutations, have been associated with activating proto-oncogene transcription in blood cancer. For instance, Herranz and colleagues identified a long-range enhancer controlled by NOTCH1 that has recurrent chromosomal duplications in human T-ALL (78). This region is activated through a mechanism of nucleosome eviction mediated by GATA3 (79), it physically interacts with the *MYC* promoter 1.4 megabases away to induce *MYC* transcription, and it has a fundamental role in the homeostasis of immature T cells. This gain-of-function amplification drives *MYC* expression downstream of NOTCH1 in T-ALL and helps to connect MYC and Notch signaling in driving oncogenesis; a different mechanism but with the same effect as the previous mentioned study in which an impaired CTCF binding (due to reduced chromatin accessibility) leads to *MYC* enhancer hijacking (44). Regarding deletions, Liu and colleagues identified a recurrent deletion of a noncoding region in T-ALL using cis-X, a computational method for identifying regulatory noncoding alterations (80). This deletion destroys the nodal CTCF binding site that forms the boundary of two independent, insulated genomic neighborhoods. As a consequence, this structural variation allows the hijacking of an active enhancer from one neighborhood by the *PRLR* promoter located in the other neighborhood, leading to PRLR upregulation and JAK2 signaling activation. In the same paper, the authors also report a recurrent intronic point mutation that activates *TAL1* oncogene transcription, a frequently mutated oncogene in T-ALL (81, 82). This point mutation creates a new active enhancer characterized by a *de novo* recognition motif for YY1. Interestingly, this transcription factor has structural properties similar to CTCF in bridging promoter–enhancer chromatin interactions (50), in addition to previous studies that show that ectopic expression of TAL1 can be also due to CTCF binding alterations that cause loss of TAD boundary insulation and subsequently enhancer hijacking (83, 84). Functional validation will be needed to better understand the complex interplay between noncoding genetic mutations, transactivation and three-dimensional chromatin organization to promote malignant transformation.

Noncoding copy number variation and point mutations are not exclusive to T-ALL. For instance, Cornish and colleagues identified recurrent mutations in B-cell lymphoma at cis-regulatory elements of naive B lymphocytes (85). Using structural data to connect cis-regulatory elements harboring structural and point mutations to promoters, they identified putative noncoding driver mutations. These mutations alter distal regulatory elements, leading to deregulation of target gene transcription. Intriguingly, they also observed that coding and noncoding mutations often converged on the same genes. For instance, MMP14 is a negative regulator of Notch signaling. It plays a key role in normal B cell differentiation, the development of diffuse large B-cell lymphoma and patient survival (86, 87). Distal cis-regulatory elements of *MMP14* are frequently deleted in B-cell lymphoma, leading to MMP14 downregulation. On top of that, coding mutations frequently affect the *MMP14* gene in several malignancies, often resulting in loss-of-function.

This convergence needs to be investigated in other contexts, for example, whether recurrent coding mutations in blood malignancies, such as in histone modifiers and chromatin remodeling factors, operate in parallel with noncoding mutations. Is there co-occurrence between the coding mutations of oncogenes and noncoding mutations at their regulatory elements in the same malignant cell or tumor type? If so, is this a synergy, an exclusion, or a redundant event? Could a cancer-related reduction of a given transcription factor level be accompanied by a reduction of its binding at regulatory elements due to mutations or epimutations at its recognition motifs? Or do tumoral cells use one or the other mechanism exclusively to silence a given transcription factor? Many questions remain open that the scientific community will have to address using the fast-growing number of methodological breakthroughs.

Noncoding loss-of-function mutations at enhancers also contribute to cancer pathology by silencing tumor-suppressor genes, although these are less well characterized. These noncoding mutations, similar to gain-of-function ones, involve different types of structural, copy number, and point mutations (**Figure 2**). A recent paper by Li and colleagues identified and validated more than two hundred tumor suppressive or oncogenic enhancers recurrently mutated in hematopoietic malignancies using enhancer CRISPR/dCas9 perturbation (activation or repression) (88). Interestingly, some of these reside in proximity to nuclear receptor–binding genomic regions, contributing to aberrant nuclear receptor signaling in blood malignancies. One example is two noncoding variants within the *PER2* enhancer that have tumor suppressive properties in AML and are potentially controlled by the nuclear receptor program. PER2 controls circadian rhythm and it has been suggested as a tumor suppressor gene (89–91). This work elegantly addresses the major challenge of prioritizing noncoding mutations and assigning functional relevance to them. Approaches like this will be needed to translate the knowledge about the noncoding genome in blood cancer into patient benefits.

## Future Perspectives

Even though compelling evidence for the contribution of noncoding mutations and epimutations to oncogenesis exists, there is still a long way to go before we can translate them into new strategies to characterize, treat and monitor blood cancer. To achieve this translational challenge, many considerations should be taken into account.

First, reliably identifying noncoding driver mutations from passenger ones remains a great endeavor due to sequencing and mapping artifacts, poorly understood mutational processes, and inaccurate estimation of the mutational background. To face these impediments, adequate statistical methods, larger datasets, higher sequence coverage, and longer and more accurate sequencing reads will be fundamental. In addition, the sequencing of normal tissues alongside malignant ones can help separate acquired from germline mutations and shed light on differentiating driver from passenger mutations.

Second, given the vastness of the noncoding genome, we need to restrict the search to relevant noncoding driver mutations. To this end, comparative genomic analysis, high-throughput *in vitro* reporter assays, and genome-wide histone modification profiling, coupled with chromatin accessibility and expression analysis, are indispensable. However, due to the highly dynamic, cell- and stimulus-specific nature of regulatory regions, it is critical to identify the right cell-type and to extend this descriptive interrogation not only to the cells of origin but also to the transformed cells.

Third, the unknown functional role of noncoding mutations imposes important limitations. To ascertain biological and mechanistic relevance, it is essential to integrate genetic and epigenetic profiling with genome conformation data and CRISPR-based functional validation. As previously described, many noncoding mutations map to enhancers, and these can exert their pathological function by altering the expression level of their target genes. However, connecting enhancers and target genes is not trivial, and due to cell-specificity and complexity, most of the associations remain unknown. Quantitative associations between noncoding variants and gene expression, and regulatory biochemical properties combined with sequencing-based chromatin conformation capture methods, such as Hi-C (24), have started providing some insights. However, reliable and reproducible identification of significant interactions between individual restriction fragments is not feasible unless Hi-C libraries are subjected to ultra-deep sequencing, which is not an economically viable solution for analyzing a comprehensive collection of cell-types or tumoral samples. To overcome it, the development of sequence-specific capturing approaches to enrich for promoter interactions and mutations in Hi-C libraries (6, 11, 69, 85), or other methods such as ChIA-PET (92) or HiChIP (93), is crucial. Nonetheless, all these methods need millions of cells, which hinders the analysis of rare cell populations such as hematopoietic stem cells or hematopoietic precursors, which are the origin cells of the majority of leukemias. Methodological breakthroughs allowing lower inputs will be fundamental in the incipient era of noncoding driver mutations.

In conclusion, the current methodological breakthroughs have positioned the scientific community in a perfect situation to explore the noncoding genome in the context of cancer. Cancer genomics is rapidly moving from a static, one-dimensional picture, to a time-dependent three-dimensional scenario to provide biological relevance of noncoding mutations at regulatory elements. We anticipate a very exciting time ahead, in which we will be fascinated by the power of noncoding mutations and epimutations in malignant transformation and the new clinical opportunities these genetic alterations will involve.

## Author Contributions

All authors listed have made a substantial, direct, and intellectual contribution to the work and approved it for publication.

## Funding

LR is funded by AGAUR project number 2019FI-B00017 of the Catalan Government (Generalitat de Catalunya). AR-M is funded by the José Carreras Leukämie-Stiftung (08R/2019). BJ is funded by FEDER/Spanish Ministry of Science and Innovation (project number RTI2018-094788-A-I00), by La Caixa Banking Foundation Junior Leader project (LCF/BQ/PI19/11690001), by the José Carreras Leukämie-Stiftung (08R/2019), and by the European Hematology Association Advance Research Grant. The funder bodies were not involved in the study design, collection, analysis, interpretation of data, the writing of this article or the decision to submit it for publication.

## Conflict of Interest

The authors declare that the research was conducted in the absence of any commercial or financial relationships that could be construed as a potential conflict of interest.
